# Continuous flow synthesis of atom-precise platinum clusters†

**DOI:** 10.1039/d4na00074a

**Published:** 2024-02-22

**Authors:** Christian Schmitt, Nicola Da Roit, Marco Neumaier, Carina B. Maliakkal, Di Wang, Thilo Henrich, Christian Kübel, Manfred Kappes, Silke Behrens

**Affiliations:** a Institute of Catalysis Research and Technology, Karlsruhe Institute of Technology Hermann-von Helmholtz-Platz 1 76344 Eggenstein-Leopoldshafen Germany silke.behrens@kit.edu; b Institute of Nanotechnology, Karlsruhe Institute of Technology Hermann-von Helmholtz-Platz 1 76344 Eggenstein-Leopoldshafen Germany; c Karlsruhe Nano Micro Facility (KNMFi), Karlsruhe Institute of Technology (KIT) Hermann-von-Helmholtz-Platz 1 76344 Eggenstein-Leopoldshafen Germany

## Abstract

Subnanometer clusters with precise atom numbers hold immense potential for applications in catalysis, as single atoms can significantly impact catalytic properties. Typically, inorganic clusters are produced using batch processes with high dilutions, making the scale-up of these processes time-consuming and its reproducibility challenging. While continuous-flow systems have been employed for organic synthesis and, more recently, nanoparticle preparation, these approaches have only rarely been applied to cluster synthesis. In a flexible, continuous flow synthesis platform, we integrate multiple continuous stirred tank reactors (CSTR) into a cascade to synthesize clusters with a precise number of atoms, demonstrating the potential of this approach for atom precise cluster synthesis and expanding the application of continuous-flow systems beyond organic synthesis.

## Abstract 2

Platinum clusters were prepared in a continuous flow synthesis using a modular platform based on a continuous stirred tank reactor cascade. Transmission electron microscopy and mass spectrometry confirmed the atom-precise cluster structure.

## Graphical abstract

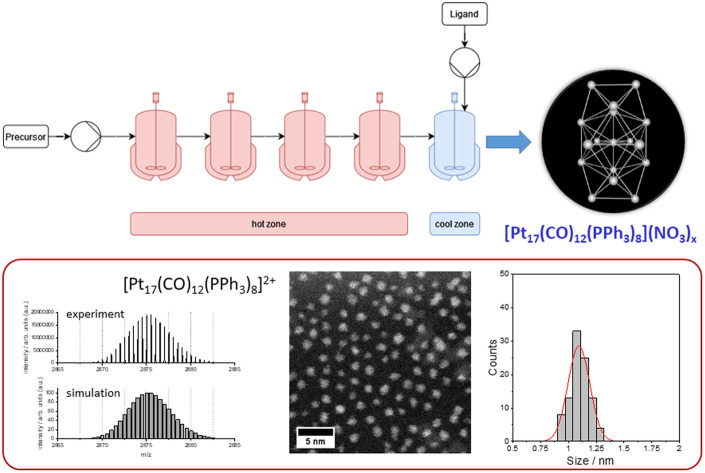

## Introduction

Platinum (Pt) is a crucially important metal for catalysis. Automotive emission control catalysts contain approx. 2 g Pt besides other platinum group metals, and Pt is also an essential component in polymer electrolyte fuel cell (PEFC) electrocatalysts for manufacturing of fuel cells. Since the total Pt resources are limited (an estimated 16 000 tons on Earth) and no base metal or non-metallic catalysts with equivalent activity and stability have yet been found as substitutes, it is essential to reduce the amount of Pt used in these and other industrial applications.^1^ Downsizing the catalyst particles to the 1 nm size range, increases the proportion of active Pt surface species and thus catalyst efficiency.^2,3^ For sizes in the sub-nanometre range (0.5–2 nm), catalytic activity no longer depends solely on particle size, but increasingly on the specific geometric and electronic structure as a cluster (or superatomic) compound.^1,4,5^ One way to tailor the mass activity of nanoscale Pt catalysts for the oxygen reduction reaction (ORR) is to control the number of active sites with an optimal coordination number per Pt mass by using cluster compounds with an atom-precise size and specific coordination geometry as catalyst precursors. For example, size-selected Pt_20_ nanoclusters achieved exceptionally high, mass-normalized activities in the ORR, when deposited at high coverage on a glassy carbon substrate.^6^ 1.1 nm Pt nanoparticles with high ORR activity were obtained using the atomically precise Pt carbonyl cluster compound [Pt_9_(CO)_18_][NBu_4_]_2_ as a precursor.^7^

Pt clusters with precisely defined numbers of atoms have attracted great interest in fundamental research. Several synthetic routes have been reported for ligand-protected Pt clusters. Carbonylation of Pt salts at atmospheric CO pressure enabled the synthesis of several Pt clusters with the overall chemical composition [Pt_3*n*_(CO)_6*n*_]^2−^ by Ciabatti *et al.*^8^ In this type of clusters, Pt_3_(CO)_6_ units are stacked in a columnar fashion, governed by metallophilic Pt–Pt interactions, steric constraints and electronic effects. For example, the pseudo-one dimensional [Pt_15_(CO)_30_]^2−^ cluster of this series already reveals a distance between the centroids of the outer Pt units of 1.2 nm.^9^ Use of phosphines (PR_3_) as additional ligands by groups of Dahl, Slovokhotov and Zacchini yielded CO/phosphine-protected Pt clusters, *e.g.* the neutral clusters^10^ [Pt_13_Au_4_(PPh_3_)_6_(CO)_10_]^0^ and [Pt_17_(PEt_3_)_8_(CO)_12_]^0^ (PEt_3_: triethylphosphine)^11^ and anionic [Pt_12_(PPh_3_)_2_(CO)_22_]^2−^ (PPh_3_: triphenylphosphine)^12^ clusters. Recently, Negishi *et al.* reported the synthesis of cationic [Pt_17_(CO)_12_(PR_3_)_8_]^*n*+^ (*n* = 1, 2) clusters.^13^ Similar to [Pt_13_Au_4_(PPh_3_)_6_(CO)_10_]^0^ and [Pt_17_(PEt_3_)_8_(CO)_12_]^0^, these clusters have the same number of metal atoms and a similar structure containing a Pt-centered, [R_3_PM–MPR_3_]-capped (with M: Pt or Au), icosahedral Pt_12_ cage. Similar structural entities were also observed for other ligand-protected metal clusters (such as [Pt_1_Ag_16_(SPhMe_2_)_8_(DPPOE)_2_]).^14,15^ However, instead of working in toxic CO atmosphere and handling under inert conditions in a multi-step procedure, the Pt cluster synthesis developed by Negishi *et al.* was much simpler, because CO was formed *in situ* by Pt-catalyzed decomposition of ethylene glycol in the presence of NaOH at elevated temperatures.^13^ Although additional Pt clusters were suggested to be formed intermittently under these conditions, they were assumed to decompose during further work-up, while only [Pt_17_(CO)_12_(PR_3_)_8_]^*n*+^ clusters (*n* = 1, 2) remained stable. Subnanometer clusters with precise atom numbers hold immense potential for application in catalysis and the prospect of tuning catalytic processes as a function of cluster size is a long-awaited goal in heterogeneous catalysis. Differences of even one atom may already cause significant changes in catalytic properties, making preparation with atomic-level precision essential.^16,17^ As shown by Heiz *et al.* for the oxidation of CO by small Pt clusters generated by high-frequency laser evaporation and soft landed on thin MgO (100) films, the catalytic activity per Pt atom is strongly cluster size dependent.^17^ Under vehicle-like conditions, the catalytic performance of the [Pt_17_(CO)_12_(PR_3_)_8_]^*n*+^ clusters (*n* = 1, 2) was evaluated for the oxidation of CO and propylene, which are the main components in automotive exhaust gases.^2^ In CO oxidation, light-off temperatures for [Pt_17_(CO)_12_(PR_3_)_8_]^*n*+^ clusters (*n* = 1, 2) were reduced from 370 to 350 °C due to their small cluster size (compared to a Pt nanoparticle reference) when the Pt_17_ clusters (or Pt reference nanoparticles) supported on γ-Al_2_O_3_ were coated on a cordierite honeycomb substrate. Pt nanoparticles (∼1 nm size) on carbon black obtained from [Pt_17_(CO)_12_(PR_3_)_8_]^*n*+^ cluster precursors were also 2.1 times more active in the oxygen reduction reaction than the commercial reference.^5^ Similarly, Pt nanoparticles with a size of ∼1.1 nm size, which was predicted to be the optimal size in a computational screening, were obtained from [Pt_9_(CO)_18_][N(butyl)_4_] in a metal–organic framework template approach and showed an activity 2.5 times higher than that of the commercial reference catalysts in the oxygen reduction reaction.^7^ Impressive resistance to sintering has been also reported for monomodal Pt clusters on Si_3_N_4_ and SiO_2_ supports at high temperatures in Ar or in the hydrogen oxidation reaction. By atomically defined clusters of precisely same size and strong support interaction, the phenomenon of Ostwald ripening (*i.e.* mass transport between particles of different size or different chemical potential) can in principle be suppressed and a better sintering stability can be achieved.^18^

**Scheme 1:**
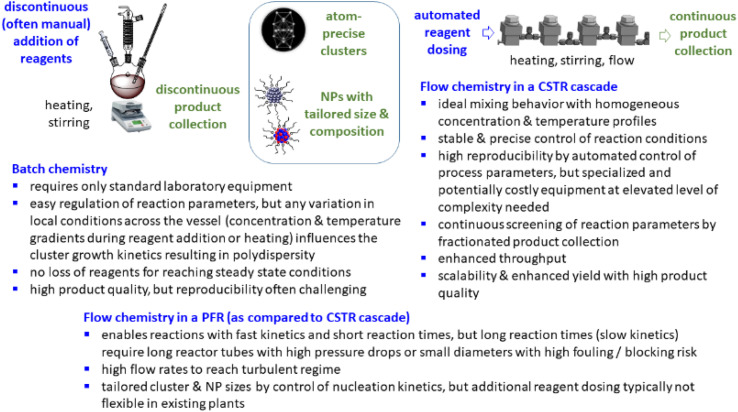
Some advantages and limitations of batch and continuous flow (in PFR and CSTR) synthesis in the preparation of clusters and nanoparticles.

Typically, inorganic clusters are produced using batch processes with high dilutions, making the scale-up of these processes time-consuming and the reproducibility challenging.^19^ Some advantages and limitations of batch and flow chemistry are illustrated in (Scheme 1). Large-scale preparation of atom-defined clusters is still in an early stage and presents immense challenges, hampering practical application. In recent years, continuous processes for nanoparticle synthesis have seen significant advancements, with plug flow reactors (PFR) and microfluidic reactors emerging as the most commonly used reactor types.^20,21^ When using strong reducing agents (*e.g.* NaBH_4_, Li naphthalenide) to produce small clusters or nanoparticles with fast reaction kinetics, inhomogeneous mixing of reactants is the main cause of polydispersity and mixing of reagents is of great importance. Microfluidic mixing units, for example, have been applied as reaction chambers for the continuous synthesis of atom-precise clusters, such as polyvinylpyrrolidone-stabilized Au_24_ clusters.^22^ Just recently, also the synthesis of ultra small IrPdPtRhRu high-entropy alloy nanoparticles by use of Li naphthalenide was reported in a continuous flow tubular reactor where the metal precursors and the reducing agent were pumped separately into the reactor and mixing occurred at the T-junction.^23^ However, these reactors are typically limited to reactions with rapid reaction kinetics,^24^ restricting the range of chemical reactions that can be employed in such systems.^24,25^ When trying to scale up batch processes using these reactors, several challenges are encountered.^26^ In PFRs, parameters such as reactor length, diameter, and flow rates determine the reaction time. For nanoparticle and cluster synthesis, where slow kinetics are often involved and reaction times in the range of several minutes to hours are required, low flow rates or extended reactor lengths are needed to achieve sufficient reaction times.^27^ This typically results either in a high pressure drop, laminar flow profiles, and/or poor mixing.^27^ Small reactor diameters in PFR often lead to fouling under these conditions, with microfluidic reactors potentially being even more susceptible to such issues.^28^ It is even more difficult to scale the synthesis of small clusters with a given number of atoms. Continuous flow, liquid phase cluster synthesis is far less explored with few examples of cluster synthesis in microfluidic or capillary reactors.^22,23,29,30^

Here we show the fully scalable, continuous flow synthesis of [Pt_17_(CO)_12_(PR_3_)_8_]^*n*+^ (*n* = 1, 2) clusters with atomic-level precision in amounts relevant to practical application. We use a modular, multistage platform based on continuous stirred tank reactors (CSTR) in a cascade that provides a simple and scalable pathway with flexible but precisely controlled reaction conditions. This method opens a general avenue for the synthesis of clusters and their application in preparative catalysis.

## Results and discussion

**Fig. 1:**
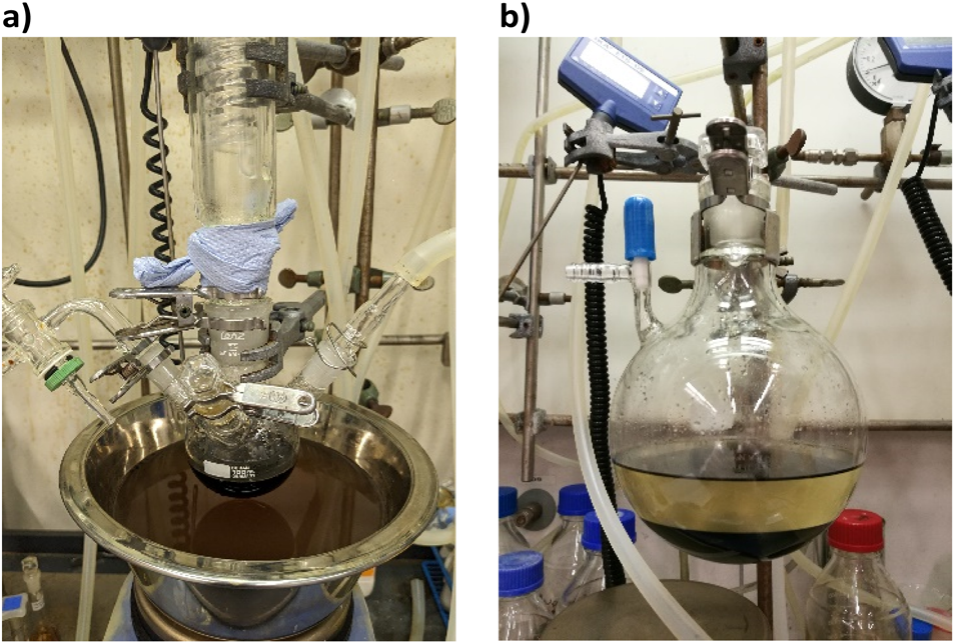
Batch synthesis of [Pt_17_(CO)_12_(PPh_3_)_8_](NO_3_)_*x*_ (*x* = 1, 2) clusters. (a) A brownish-black color of the reaction mixture indicates cluster formation. (b) The clusters were isolated from the reaction mixture by extraction in dichloromethane (black bottom phase).

The cluster synthesis was based on a batch procedure originally developed by Negishi *et al.*, which was modified here for continuous cluster synthesis.^13^ The synthesis of [Pt_17_(CO)_12_(PPh_3_)_8_]^*n*+^ (*n* = 1, 2) clusters is quite simple in the batch procedure and can be carried out in NaOH/ethylene glycol solution from commercial reagents, avoiding the use of toxic CO atmosphere. Thus, this reaction seems to be ideally suited to study the synthesis of clusters in continuous flow. The reaction scheme is illustrated in Fig. 1. Specifically, the solution of Pt(NO_3_)_2_ and NaOH in ethylene glycol was heated in a glass flask at 120 °C, which initiated the reduction of Pt^2+^ ions and the generation of CO by oxidation of ethylene glycol. After 45 min of reaction time, the colour of the reaction mixture changed to brownish black indicating reduction and cluster formation.

**Fig. 2:**
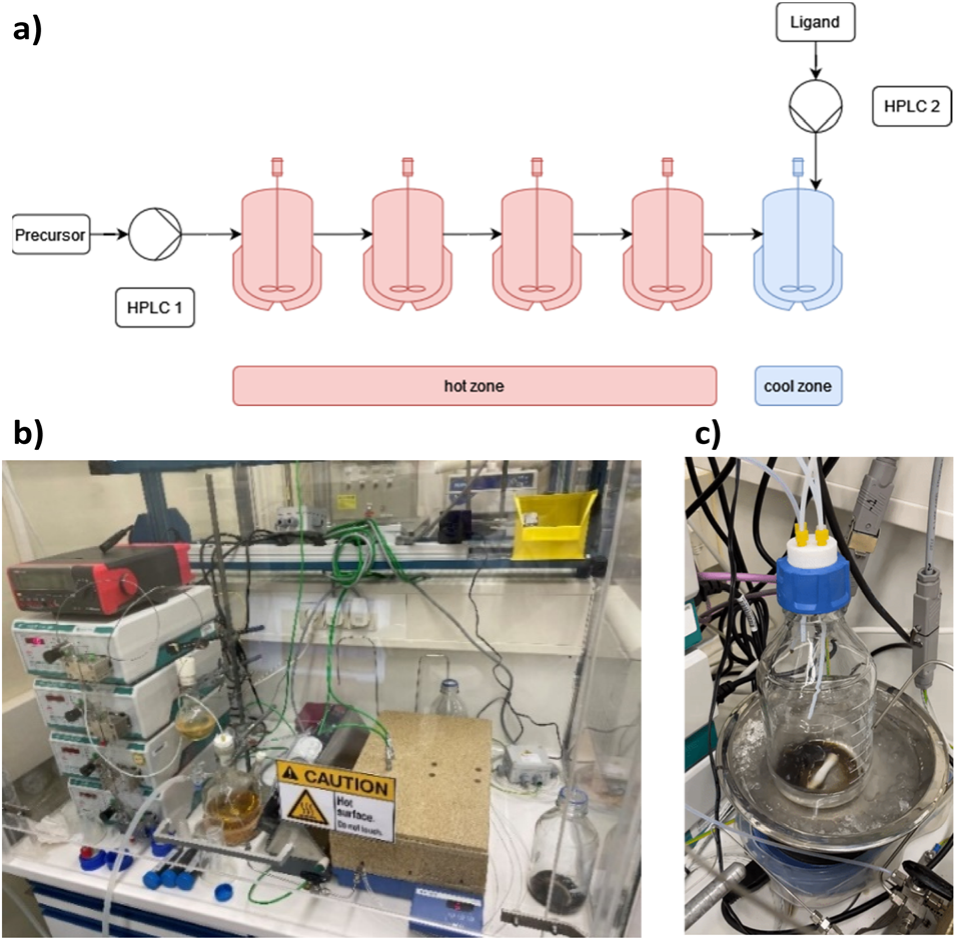
(a) Schematic representation of the CSTR module. Photos of (b) the modular laboratory reactor and (c) the continuously collected cluster product.

Then, a solution of PPh_3_ in acetone was added and the PPh_3_-stabilized clusters were extracted in dichloromethane. After removal of the solvent in vacuum, subsequent steps of washing yielded the [Pt_17_(CO)_12_(PPh_3_)_8_]^*n*+^ clusters as a black powder. For the continuous flow cluster synthesis, a laboratory plant with a CSTR cascade was employed (Fig. 2). The synthesis platform was designed modularly with multiple CSTRs of variable volume that can be operated either as a cascade or in parallel using up to three different heating zones. While batch cluster synthesis can be easily carried out using standard laboratory equipment (Fig. 1), continuous flow synthesis requires specialised and more costly equipment with a higher degree of complexity. However, the advantages of continuous flow synthesis are increased reproducibility of high quality cluster products through automated and precise control of reaction parameters and stable, steady-state reaction conditions, and high yield due to continuous flow operation, with the possibility of scaling up to industrial levels in principle (Scheme 1). While a PFR exhibits kinetics similar to batch synthesis over its entire length, achieving ideal PFR behaviour requires a turbulent flow regime and thus, either high flow rates or small diameters. This presents practical challenges, such as the need for very long reactors to achieve the required residence time and flow rate in the present case (*e.g.* approx. 80 m for 40 min reaction time). Tubular reactors of this length result in a high-pressure drop across the reactor, particularly for higher viscosity solvents, which has several drawbacks. On the other side, microfluidic PFR reactors with small diameters may suffer significantly from fouling. This is particularly important when transferring the synthesis process from batch to continuous flow, where the various reaction conditions need to be optimized and adapted. During this exploratory phase, the microfluidic channels may be blocked by formation of particulate by-products or product deposition on the reactor walls under non-ideal reaction conditions. Overall, a (microfluidic) PFR appeared to be less suitable for use in the present synthesis of Pt clusters. It should be also noted that the choice of reactor geometry was beyond the present Pt cluster synthesis and considered in a more general context of nanoparticle and cluster synthesis with slow reaction kinetics. The synthesis platform based on a CSTR cascade is highly flexible, where the number, volume and arrangement of the individual reactors can be easily adapted to the specific reaction conditions for different synthesis procedures in an exploratory manner.

For the procedure described herein, a set-up with two temperature zones was employed, *i.e.* a high-temperature zone (hot zone) and a low-temperature zone (cool zone). Four CSTRs (10 mL volume each) were operated in a cascade, which could be heated up to 300 °C. Finally, the reaction was quenched and the product collected in a stirred tank reactor (glass bottle) where a solution of the PPh_3_ ligands in acetone was added.

**Fig. 3:**
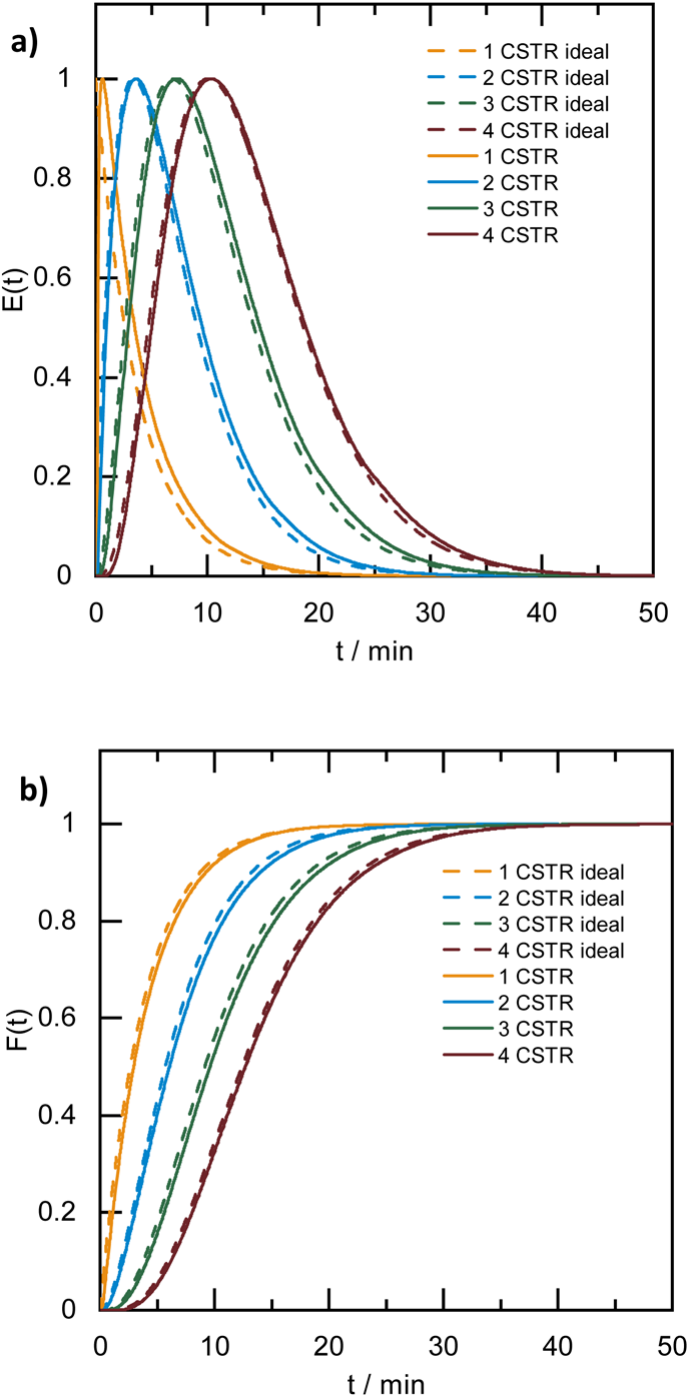
RTD profiles of the CSTR cascade (for a one to four CSTR configuration) (a) *E*(*t*) and (b) *F*(*t*) (flow rate: 3 mL min^−1^, stirring speed: 650 rpm, solid lines: experimental RTD profiles, dashed lines: profiles based on the ideal CSTRs in series model).

A broad residence time distribution generally might have a negative effect on conversions and/or selectivities in syntheses, and specifically lead to a large size distribution in nanoparticle/cluster syntheses. To assess the mixing performance and the residence time distribution of the CSTR reactors, the outlet concentration profiles were measured and compared to the RTD of the ideal CSTR series model (eqn (1)) (for experimental set-up see ESI, Fig. S2†), where *t* is the time (with *t*_0_ time of tracer injection), *n* the numbers of CSTRs in series and *τ* the residence time.^25,31,32^Fig. 3 depicts the RTD *E*(*t*) and residence time sum curve *F*(*t*) for 1 to 4 CSTR in series, as compared to the ideal profiles.1
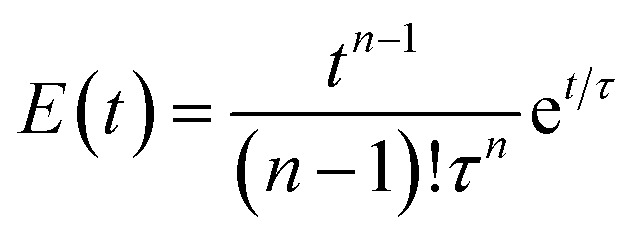


In the CSTR module of the plant, the tailing of the outlet concentration profile decreased as the number of CSTRs in the cascade was increased from one to four CSTRs, as the CSTR module approaches the behavior of the plug flow reactor.^32^ The good performance of the CSTR module is critical to achieve a uniform product. In addition, the outlet concentration profiles were studied for a cascade of five CSTRs increasing the flow rates from 1.0 to 5.0 mL min^−1^ while keeping the stirring speed constant at 650 rpm. The mixing behavior of the CSTRs was not affected by changing the flow rates (see ESI, Fig. S3†).

When the stirring speed (flow rate 3 mL min^−1^) exceeded 100 rpm, the outlet concentration profile was also unaffected by the stirring speed (see ESI, Fig. S4†). It was only when the stirrer was switched off that ideal mixing of the reactors did not occur (flow rate 3 mL min^−1^) and CSTR behaviour was not observed. Given the small volume of the reactors, the volume of the stirring bars should be taken into account, resulting in a reactor volume of 9.5–9.8 mL. Therefore, for flow rates in the range of 1.0 to 5.0 mL min^−1^ and stirring speeds >100 rpm, homogeneous stirring of the individual CSTRs by the multipoint magnetic stirrer and near ideal CSTR cascade behaviour can be assumed. Depending on the flow rate, the total residence time and therefore reaction time can be varied from approximately 8 to 40 minutes with good CSTR performance. After characterizing the CSTR module, we demonstrated the potential of our continuous flow synthesis platform for the scale-up of atom-precise clusters using the example of [Pt_17_(CO)_12_(PPh_3_)_8_]^*n*+^ (*n* = 1, 2) cluster synthesis. Four CSTRs with a reactor volume of 10 mL each were operated in the cascade (Fig. 2). For the four CSTR configuration and a flow rate of 3 mL min^−1^, the average residence time was 13.3 min. For continuous cluster synthesis, one of the glass storage vessels was charged with the solution of the Pt(NO_3_)_2_ precursor and NaOH in EG. The CSTRs were then heated to 120 °C while EG was introduced *via* the HPLC-1 pump. After reaching the reaction temperature of 120 °C, the precursor solution was added (flow rate 3 mL min^−1^) and steady-state conditions were reached after approximately 40 min. The reaction mixture was finally collected at the outlet of the CSTR module in an argon flushed glass bottle and cooled on an ice bath while stirring. Quenching the reaction at low temperatures was a prerequisite to prevent the formation of clusters of larger sizes and thus obtain a uniform product.^5^ A solution of the triphenylphosphine ligand in acetone was continuously added to the brownish-black reaction product *via* the HPLC-2 pump while stirring under argon (Fig. 2). After isolation and drying under vacuum (see Experimental section), [Pt_17_(CO)_12_(PPh_3_)_8_]^*n*+^ (*n* = 1, 2) was received as a black powder (yield 0.3307 g).

**Fig. 4:**
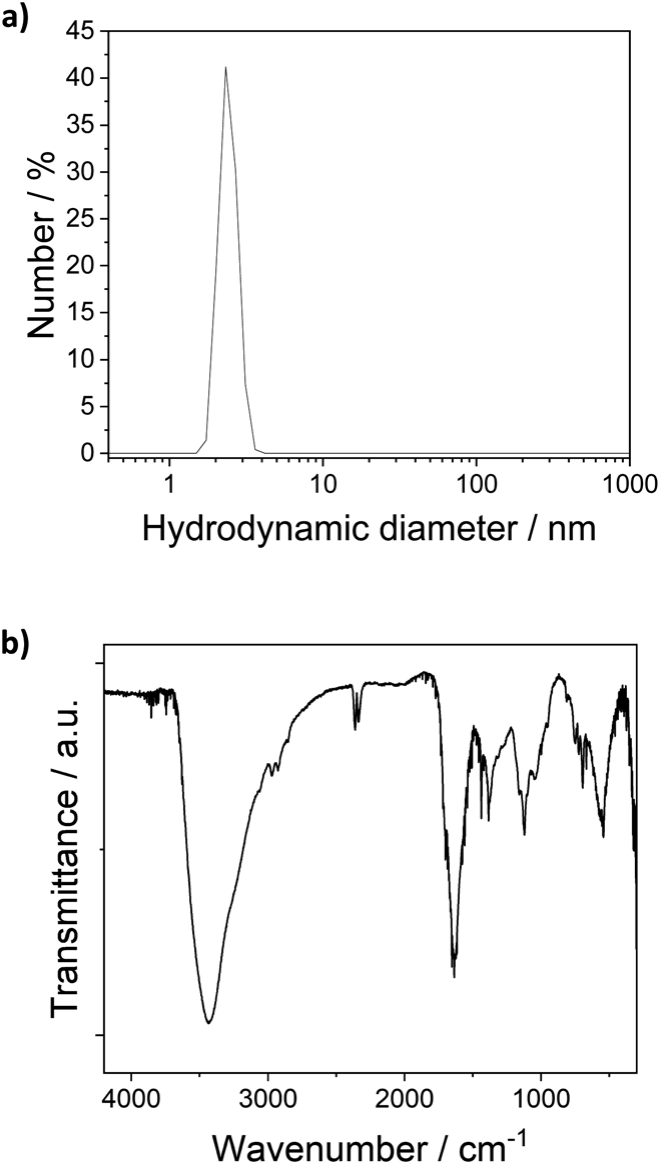
(a) DLS analysis of the [Pt_17_(CO)_12_(PPh_3_)_8_]^*n*+^ (*n* = 1, 2) clusters in dichlormethane (hydrodynamic diameter 2.4 nm). (b) FT-IR spectra of [Pt_17_(CO)_12_(PPh_3_)_8_](NO_3_)_*x*_ (*x* = 1, 2) clusters.

**Fig. 5:**
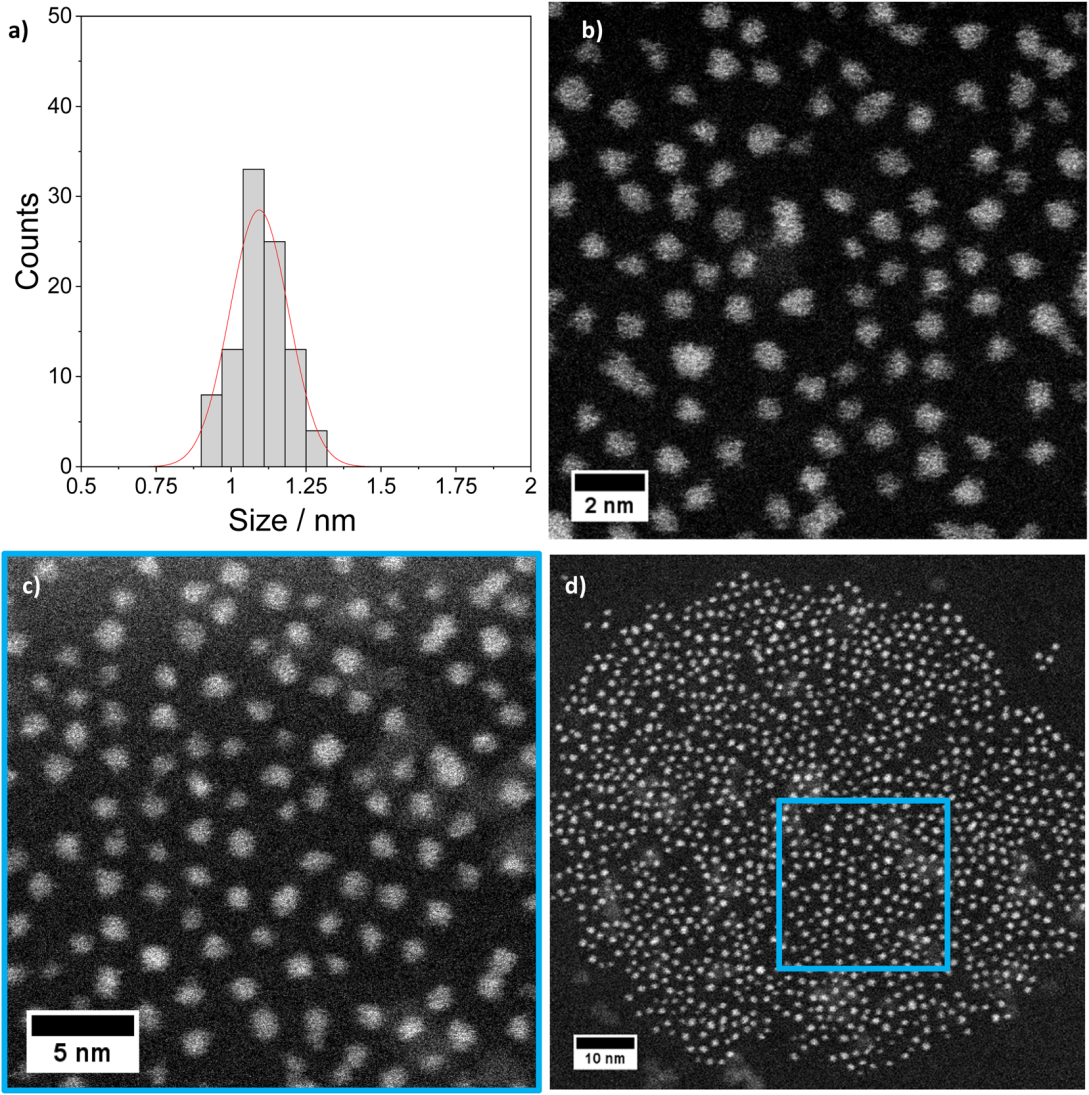
STEM imaging: (a) size histogram of [Pt_17_(CO)_12_(PPh_3_)_8_](NO_3_)_*x*_ (*x* = 1, 2) obtained by analysing low magnification LN_2_ cryo HAADF-STEM images (mean size: 1.1 ± 0.1 nm). (b)–(d) Higher magnification STEM micrographs of the same clusters imaged at room temperature (details in ESI Fig. S5 and S6†).

The [Pt_17_(CO)_12_(PPh_3_)_8_]^*n*+^ (*n* = 1, 2) cluster product was characterized by transmission electron microscopy (TEM), dynamic light scattering (DLS), and mass spectrometry (MS). Based on DLS measurements, the hydrodynamic diameter of the clusters is 2.4 nm (Fig. 4a). Fig. 5 shows HAADF-STEM images of the [Pt_17_(CO)_12_(PPh_3_)_8_]^*n*+^ clusters measured at room temperature. It should be noted that the [Pt_17_(CO)_12_(PPh_3_)_8_]^*n*+^ (*n* = 1, 2) clusters have an ellipsoidal shape according to single crystal XRD analysis and their orientation may vary on the TEM grid. The cluster size and size distribution were determined from HAADF-STEM images by statistical measurement of the major axis from a large number of clusters. Low magnification images and cryo conditions were used to measure the cluster size to avoid damage and restructuring of clusters under the electron beam (see ESI, Fig. S5†). The mean diameter based on the major axis was 1.1 ± 0.1 nm.

The clusters were further investigated by FT-IR spectroscopy of the finely powdered cluster sample pressed in KBr discs. The spectra showed several bands of the PPh_3_ ligands (3060 cm^−1^, 1465 cm^−1^, 1437 cm^−1^, 1099 cm^−1^, 997 cm^−1^, 748 cm^−1^) (Fig. 4b),^13^ confirming the presence of the PPh_3_ ligands in the cluster product. CO bands were not observed in the FTIR spectra of the sample in KBr discs or in FTIR-ATR spectroscopy. Given the synthesis conditions, the anion in the cluster product was expected to be a nitrate ion (NO_3_^−^). The cluster anion reported previously for the synthesis by Negishi *et al.* was chloride, which is also a well-known catalyst poison in many catalytic reactions, and the majority of the cluster product (80%) was monocationic.^13^ Bands observed in the FTIR spectra of the cluster at 835 cm^−1^, 1355/1383 cm^−1^ and 694/668 cm^−1^ could be attributed to the NO_3_^−^ anion (Fig. 4b). For [Pt_17_(CO)_12_(PPh_3_)_8_](NO_3_)_*x*_, the nitrogen content of the cluster product obtained by elemental analysis was 0.2 wt%, which is in good agreement with the 0.014 wt% and 0.027 wt% nitrogen that are expected for [Pt_17_(CO)_12_(PPh_3_)_8_](NO_3_) and [Pt_17_(CO)_12_(PPh_3_)_8_](NO_3_)_2_, respectively. This would suggest a 1 : 1 mixture of the divalent and monovalent clusters in the sample; however, an exact interpretation is difficult due to the error of ICP-OES analysis.

**Fig. 6:**
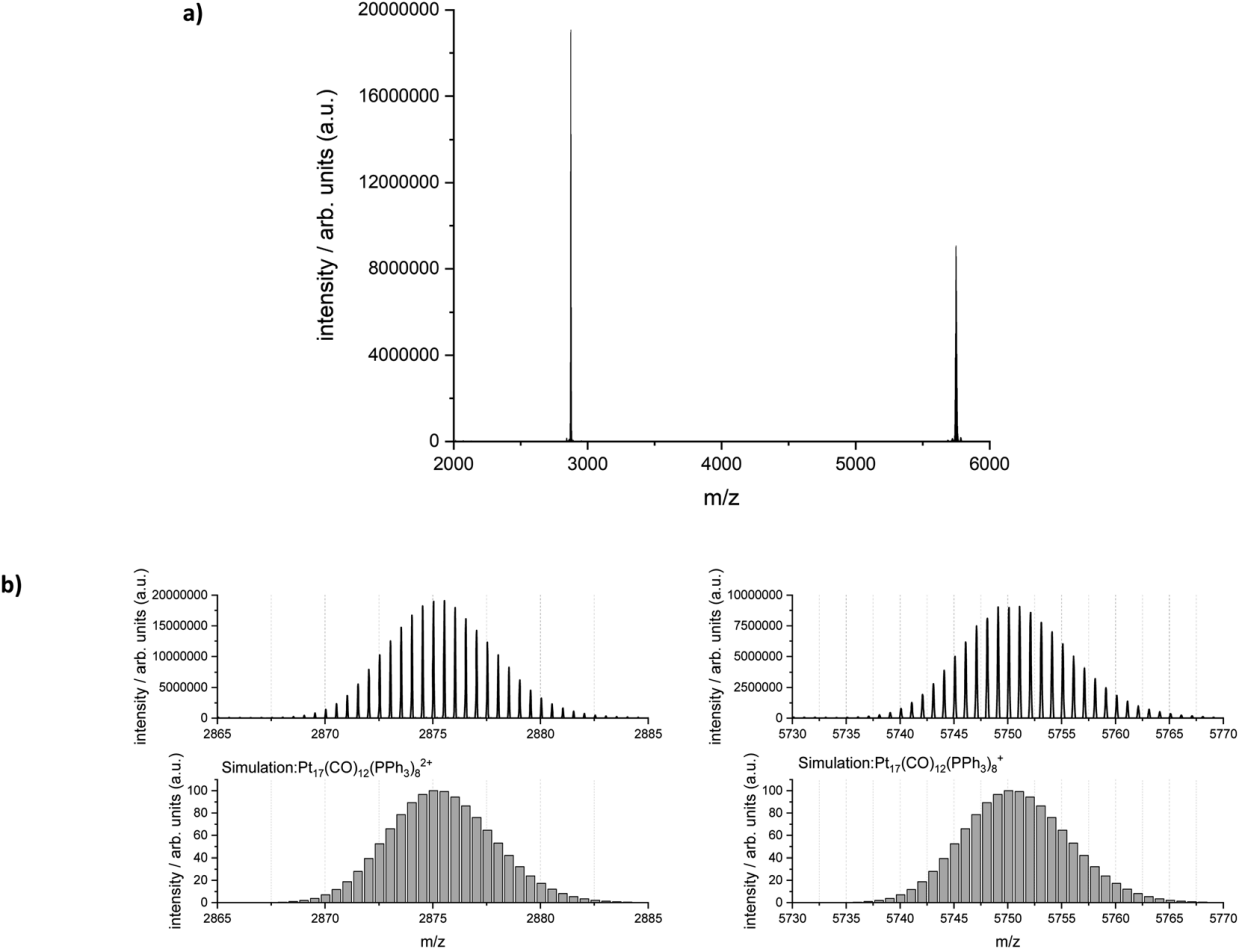
(a) ESI-MS spectra of the isolated [Pt_17_(CO)_12_(PPh_3_)_8_]^*n*+^ (*n* = 1, 2) clusters. (b) Experimental and simulated mass spectra of the main reaction product [Pt_17_(CO)_12_(PPh_3_)_8_]^*n*+^ (*n* = 1, 2). The spectrum was taken with an Orbitrap Exploris 2040 mass spectrometer.

Electrospray ionization-mass spectrometry (ESI-MS) was further used to characterize the reaction product. Fig. 6 shows the measured and simulated isotopic distributions of [Pt_17_(CO)_12_(PPh_3_)_8_]^2+^ at *m*/*z* = 2875.03 and [Pt_17_(CO)_12_(PPh_3_)_8_]^+^ at *m*/*z* = 5750.07. The spectra were recorded after purifying the product by six washing steps. The ESI-MS spectra demonstrate that the atom-precise [Pt_17_(CO)_12_(PPh_3_)_8_]^*n*+^ (*n* = 1, 2) cluster was formed by continuous flow synthesis in four CSTRs and could be isolated after several steps as a pure product. The ESI-MS results confirmed the presence of 12 CO ligands on the Pt_17_ cluster core.

The crude reaction product also contained some [Pt_19_(CO)_13_(PPh_3_)_9_]^2+^ clusters as a by-product (Fig. S1†), which were removed during the purification process (see Experimental section). When the reaction temperature of the CSTR cascade was further increased to 125 °C during continuous flow synthesis, however, the main reaction product was [Pt_19_(CO)_13_(PPh_3_)_9_]^2+^ instead of [Pt_17_(CO)_12_(PPh_3_)_8_]^*n*+^ (*n* = 1, 2) (Fig. S6†). Thus, the reaction temperature seems to be a crucial parameter in the formation of the clusters. This opens a promising avenue for exploring Pt clusters with different nuclearities by controlling the process parameters (*e.g.* reaction temperature). However, achieving a pure product of individual cluster species may require not only optimised reaction temperatures, but also further developments with respect to their stabilisation, including the appropriate choice and concentration of ligands, as well as a purification process tailored to the desired cluster nuclearity. The influence of temperature and other reaction parameters (such as the type of ligands) on the formation of clusters of different nuclearities will be the subject of future investigations.

It is interesting to note that if diphenyl(*p*-tolyl)phosphine (PPh_2_(*p*-tol)) was used as ligand instead of PPh_3_, [Pt_19_(PPh_2_(*p*-tol))_9_(CO)_13_]^2+^ was also previously observed together with [Pt_17_(PPh_2_(*p*-tol))_8_(CO)_12_]^*n*+^ (*n* = 1 or 2).^1^ The batch synthesis of an anionic [Pt_19_(CO)_21_(NO)]^3−^ cluster was also reported by Longoni *et al.*^33^ The clusters were crystallized from the solution of the [Pt_19_(CO)_22_]^4−^ clusters and NOBF_4_.^33,34^ In the future, it will be interesting to explore relative redox activity of Pt_17_*vs.* Pt_19_ clusters, also as a function of charge state and electronic structure.^35^

## Experimental

### Materials

Ethylene glycol (EG, Thermo Scientific, 99+% extra pure), acetone (Thermo Scientific, 99+% extra pure), methanol (Sigma-Aldrich, 99.8%), toluene (Thermo Scientific, ≥99.8%), Pt(NO_3_)_2_ (Evo Chem Advanced Materials, 99.95%), triphenylphospine (Thermo Scientific, 99+%), dichloromethane (Thermo Scientific, 99.6%).

### Reactor design

For the synthesis of the clusters, a continuous flow, modular laboratory plant was designed, which was equipped with multiple CSTRs of flexible reactor volume. In this study, four stainless steel CSTRs with a volume of 10 mL each were used for continuous flow synthesis. The CSTRs were embedded in an aluminum block for good and uniform heat transfer, which was equipped with four heating cartridges (Horst GmbH, max. 200 W each). A multi-point magnetic stirrer (RO 15 from IKA-Werke GmbH) with individual, Teflon-coated stirring bars in each of the CSTRs was used for stirring and ensured good mixing of the reaction mixture in the reactors. An additional jacket of Vermiculite^®^ (K. Hoffmann GmbH) was used for thermal insulation. The solutions of precursors and ligands were introduced into the reactor cascade with high-performance liquid chromatography (HPLC) dosing pumps (Bischoff Analysentechnik und -geräte GmbH).

### RTD measurements

The residence time distribution (RTD) of the reactors was determined by conductometry using aqueous NaOH (1 mL, 1 mol L^−1^) as a tracer. Distilled water was pumped through the CSTR cascade using a HPLC dosing pump. Typical volumetric flow rates were 1 to 5 mL min^−1^. To monitor the time-dependent ion concentrations, the liquid was passed through a capillary flow cell (Malvern Panalytical) with an external voltage of 8.5 V (power supply: Manson NSP3630, 1 36 V/DC 3 A max).

Current [*I*] = μA and voltage [*U*] = V were recorded over time by a multimeter (TrueRMS bench multimeter UT803, UNI-TREND Technology). The resistance *R* was calculated from *I* and *U* using Ohm's law. To obtain the residence time distribution (RTD), the dead volumes of the tubes were subtracted and the curves were normalized between 0 and 1. The RTD of 1 to 4 reactors in the cascade was determined for different volumetric flows.

### Batch synthesis of [Pt_17_(CO)_12_(PPh_3_)_8_](NO_3_)_*x*_ (*x* = 1, 2) clusters

[Pt_17_(CO)_12_(PPh_3_)_8_](NO_3_)_*x*_ (*x* = 1, 2) clusters were synthesized using a modified batch procedure, previously described by Negishi *et al.*^13^ All solutions were degassed in vacuum prior to use, and the reaction was carried out under argon atmosphere. Briefly, a solution of Pt(NO_3_)_2_ (0.1595 g, 0.5 mmol) in EG (25 mL) was injected in a solution of NaOH (0.4001 g, 10 mmol) in EG (100 mL) at 120 °C while stirring. After 45 min of reaction at 120 °C, the color of the reaction mixture changed to brownish black indicating reduction and cluster formation. The reaction mixture was then cooled to room temperature with an ice bath. Triphenylphosphine (PPh_3_) (2.6225 g, 10 mmol) in acetone (50 mL) was added and the mixture stirred for 30 min. The clusters were extracted in dichloromethane using a mixture of dichloromethane/water (1 : 1). After removal of the solvent in a rotary evaporator (100–200 mbar, 30 °C), the sample was washed several times with methanol/water. The clusters were purified by sixfold dissolution and precipitation in dichloromethane and hexane. Finally, the [Pt_17_(CO)_12_(PPh_3_)_8_](NO_3_)_*x*_ (*x* = 1, 2) clusters were isolated as a black powder after drying in vacuum (<20 mbar; 30 °C) overnight (yield: 0.0397 g, 26%).

### Continuous flow synthesis of [Pt_17_(CO)_12_(PPh_3_)_8_](NO_3_)_*x*_ (*x* = 1, 2) clusters

For the continuous flow cluster synthesis, the modular synthesis platform with four CSTRs in series and an additional glass bottle for cooling and collection of the cluster product were used. For this purpose, EG was degassed in vacuum at 60 °C for 30 min while stirring. Pt(NO_3_)_2_ (0.6385 g, 2.5 mmol) and NaOH (1.6005 g, 40 mmol) were dissolved in 500 mL EG, degassed in vacuum for 60 min at room temperature (RT), and transferred to the storage container under argon. Triphenylphosphine (PPh_3_) (8.3933 g, 32 mmol) was dissolved in acetone (160 mL) and transferred to a second storage container under argon. Initially, the reactors were flushed with EG and gas bubbles remaining in the reactors were removed. The reactors were heated to 120 °C. During heating, the flow rate of EG was 3 mL min^−1^ (HPLC-1). After reaching the reaction temperature of 120 °C, the solution of Pt(NO_3_)_2_ and NaOH in EG was added with a flow rate of 3 mL min^−1^. After reaching steady state conditions (approx. 40 min), the reaction product was collected in an argon-flushed glass bottle and cooled while stirring. The solution of the PPh_3_ ligand was continuously dosed to the reaction product (1.2 mL min^−1^, HPLC-2). To isolate the clusters from the caustic EG reaction mixture, they were extracted in dichloromethane using a mixture of dichloromethane/water (1 : 1). Dichloromethane was removed in a rotary evaporator (100–200 mbar, 30 °C). The residue was purified six times by suspending it in methanol/water followed by centrifugation (7197 rcf). The clusters were purified by sixfold dissolution and precipitation in dichloromethane and hexane. Finally, the [Pt_17_(CO)_12_(PPh_3_)_8_](NO_3_)_*x*_ (*x* = 1, 2) clusters were isolated as a black powder after drying in vacuum overnight (<20 mbar; 30 °C) (yield: 0.331 g, 32%).

### Characterization

The Pt content was determined by inductively coupled plasma optical emission spectroscopy (ICP-OES) (Agilent 725 ICP-OES spectrometer) after dissolution of the clusters in aqua regia. SEM-EDX analysis was performed on a Gemini SEM 500 from Zeiss GmbH equipped with a Schottky field emission cathode. For dynamic light scattering (DLS) analysis, a Zetasizer Nano ZS (Malvern Instruments, 633 nm, non-invasive backscattering at 173°) was used and the sample diluted in dichlormethane prior to DLS measurement in quartz cuvettes (10 mm, Hellma Analytics, Germany). For FT-IR analysis, finely powdered cluster samples were pressed into discs with oven dried, spectroscopic-grade KBr and measured in the FT-IR spectrometer (Agilent Technologies Varian 660) in transmission mode (resolution 2 cm^−1^). High-resolution high-angle annular dark-field (HAADF) scanning transmission electron microscopy (STEM) imaging was carried out on Themis 300 and Themis Z transmission electron microscopes (TEM) (Thermofisher Scientific), both equipped with a probe aberration corrector and operated at 300 kV. For TEM measurements, small droplets of the cluster solution in dichloromethane were placed on hydrophilized amorphous carbon-coated Cu grids and dried in air. To reduce damage by the electron beam, some TEM measuremets were performed close to liquid N_2_ temperature using a Gatan 915 cryo TEM holder. The mean particle diameter was calculated from relatively low-magnification HAADF-STEM images (640 k, pixel size 0.06921 nm) by measuring the size of at least 100 particles. The clusters were further characterized by high resolution mass spectrometry (MS) using a commercial Thermofisher Scientific Orbitrap Exploris 240 MS and a Bruker timsTOF-MS both equipped with an electrospray ion source (ESI). TIMS is a high resolution variant of ion mobility spectrometry allowing isomer separation based on collision cross section. For this study the timsTOF was operated in the “TIMS-off” mode (*i.e.* without isomer separation), as a high resolution ESI-MS with a nominal mass resolution of *ca.* 50 000. The following ESI source conditions were typically used for measurements of clusters with the timsTOF-MS: sample concentration 40–100 nmol L^−1^ in toluene/methanol (1 : 1), flow rate 3 μL min^−1^, capillary voltage 4.5 kV, nebulizer gas 0.3 bar, dry gas 2 L min^−1^ and dry gas temperature 300 °C. Source conditions of the Orbitrap MS were typically: capillary voltage 3.2–3.8 kV, sheath gas 5, aux gas 5 and sweep gas 2 (arb. units), ion transfer tube temperature 320 °C.

## Conclusions

Due to their defined atomicity, geometric and electronic structures, cluster compounds may exhibit high atom-dependent catalytic activity and high stability against sintering, which allows, *e.g.* to reduce the use of noble metals in catalysis. While solution chemistry developed over the last decades offers various synthetic approaches for ligand-stabilized cluster compounds, access to clusters with well-defined nuclearity on a preparative scale is still an immense challenge. Herein, we present a flexible platform for the synthesis of cluster compounds in continuous flow. The platform consists of multiple CSTR reactors, which may be operated in series (cascade) or in parallel. Ideal mixing behavior with homogeneous concentration and temperature profiles was achieved by strong stirring in each CSTR. By using multiple CSTRs in a cascade, the behavior of the platform approached the ideal plug flow reactor with narrow residence time distribution. This is a prerequisite for high conversion and selectivity in any chemical reactions, but especially in nanoparticle or cluster syntheses for achieving narrow size distributions. The potential of the continuous flow synthesis platform for the scale-up of atom-precise clusters was demonstrated for the synthesis of [Pt_17_(CO)_12_(PPh_3_)_8_]^*n*+^ (*n* = 1, 2) clusters. After working up the reaction product, the [Pt_17_(CO)_12_(PPh_3_)_8_](NO_3_)_*x*_ (*x* = 1, 2) could be isolated as a pure cluster compound. The atom-precise composition of the sample was shown by ESI mass spectrometry, transmission electron microscopy, and dynamic light scattering. ICP-OES and FTIR analysis further confirmed the presence of nitrate as counter ion(s) of the mono- or divalent cluster cation. If the reaction temperature was further raised from 120 °C to 125 °C, the major reaction product was [Pt_19_(CO)_13_(PPh_3_)_9_]^2+^ in addition to some [Pt_17_(CO)_12_(PPh_3_)_8_]^*n*+^ clusters. This opens up a promising avenue for atom-precise clusters with different nuclearities by further optimizing the reaction conditions (*e.g.*, temperature, ligands, *etc.*). This will be a subject of future studies. In this context, the implementation of analytics for on-line monitoring of precursor consumption and/or cluster formation could contribute to a better understanding of the formation kinetics and mechanisms, and would allow better control of the formation processes in the future. In addition, the continuous flow synthesis is a suitable tool to scale the cluster product to larger quantities, *i.e.* the yield of the cluster compounds can be scaled to 1 g [Pt_17_(CO)_12_(PPh_3_)_8_](NO_3_)_*x*_ in 10 h of reaction. Thus high cluster yields may be achieved due to continuous flow operation, which could be scaled to industrial levels in principle. It should be noted that the potential of the modular synthesis platform goes beyond the current synthesis of Pt clusters and, due to its high modularity and flexibility, it is interesting for implementing the synthesis of a plethora of different cluster compounds and nanoparticles. As a conclusion, the continuous flow platform with multiple CSTRs in cascade is a highly promising synthetic approach for future development of various cluster syntheses, especially when larger quantities are required for application, *e.g.* in catalysis.

## Data availability

TEM, MS, DLS, and IR data included in this manuscript, along with metadata, are available at KITopen/RADAR (DOI: 10.35097/1955).

## Author contributions

The manuscript was written through contributions of all authors: C. Schmitt designed the synthesis platform, carried out the continuous flow cluster synthesis and characterization, and wrote the original draft; N. Da Roit performed the batch cluster synthesis and standard TEM characterization; C. Mailikkal, D. Wang, C. Kübel carried out HAADF STEM investigations on the clusters; M. Neumaier and M. Kappes characterized the clusters by mass spectrometry; T. Henrich provided technical support with the synthesis platform; S. Behrens conceptualized the synthesis platform/experiments, wrote and revised the manuscript. All authors have given approval to the final version of the manuscript.

## Conflicts of interest

There are no conflicts to declare.

## Supplementary Material

NA-006-D4NA00074A-s001
