# Supplementary material for: Continuous flow synthesis of atom-precise platinum clusters
Source: Nanoscale Adv. 2024 Feb 22;6(9):2459–68. doi: 10.1039/d4na00074a (PMC11059489; doi:10.1039/d4na00074a)
Supplement: NA-006-D4NA00074A-s001 [file NA-006-D4NA00074A-s001.pdf]

Electronic Supplementary Material (ESI) for Nanoscale Advances.
This journal is © The Royal Society of Chemistry 2024

Supporting Information

Continuous flow synthesis of atom-precise platinum clusters

Christian Schmitt,[a] Nicola Da Roit,[a] Marco Neumaier,[b] Carina B. Maliakkal,[b] Di Wang,[b,c] Thilo Henrich,[a] Christian Kübel,[b,c] Manfred Kappes,[b] Silke Behrens[a,] *

[a] Institute of Catalysis Research and Technology Karlsruhe Institute of Technology, Hermann-von Helmholtz-Platz 1, 76344 Eggenstein-Leopoldshafen, Germany

[b] Institute of Nanotechnology Karlsruhe Institute of Technology, Hermann-von Helmholtz-Platz 1, 76344 Eggenstein-Leopoldshafen, Germany

[c] Karlsruhe Nano Micro Facility (KNMFi), Karlsruhe Institute of Technology (KIT), Hermann-von-Helmholtz-Platz 1, 76344 Eggenstein-Leopoldshafen, Germany

*Corresponding author: silke.behrens@kit.edu

CONTENT

Page

Figure S1. ESI- MS spectra of the reaction product at 120°C prior to purification. The major product observed was $[Pt_{17}(CO)_{12}(PPh_3)_8]^{n+}$ (n = 1, 2), with some $[Pt_{19}(CO)_{13}(PPh_3)_9]^{2+}$ as a minor by-product. The spectrum was taken with a timsTOF-MS. S4

Figure S2. RTD measurement: A conductivity flow cell was used to record time-dependent concentration profiles at the outlet of each CSTR. S5

Figure S3. RTD profiles of five CSTRs in a cascade for different flow rates a) $E(\Theta)$ and b) $F(\Theta)$ (flow rates: 1 - 5 mL min^{-1}, stirring speed: 650 rpm; solid lines: experimental RTD profiles, dashed lines: RTD of the ideal CSTRs in series model, all normalized with $\Theta=t/\tau$). S5

Figure S4. RTD profiles of five CSTRs in a cascade for different stirring speeds a) $E(t)$ (stirring speed: 0 and 650 rpm, flow rate: 3 mL min^{-1}, solid line: experimental RTD profile, triangles : RTD of the ideal CSTRs in series model). S6

Figure S5. Room temperature STEM imaging: HAADF-STEM images of the $[Pt_{17}(CO)_{12}(PPh_3)_8]^{n+}$ (n = 1, 2) clusters showing the same area of the sample, but with some clusters merged in (b) due to damage caused by the electron beam. In (a), the region shown is cropped from an image captured at lower magnification and thus a lower dose rate ($0.38\cdot10^9$ electrons/sÅ^2). Image in (b) is acquired after the image in (a), and at a higher magnification and higher dose rate ($1.53\cdot10^9$ electrons/sÅ^2). Some of the clusters, S7

marked here in red, have merged in the latter image. Such merging can in turn lead to an overestimation of the cluster size. In order to decrease the effect of the electron beam and to more accurately measure the cluster dimensions, we also imaged the sample in a cryogenic holder as shown in the next image.

Figure S6. Low temperature STEM imaging: Low magnification HAADF-STEM images of the $[Pt_{17}(CO)_{12}(PPh_3)_8]^{n+}$ clusters, in a cryo holder at around -185 ºC, which were used for determination of the cluster size; mean size 1.09 ± 0.10 nm. S8

Figure S7. Experimental ESI-MS spectra of the reaction product after continuous flow synthesis at 125°C. Experimental and simulated ESI-MS spectra of $[Pt_{19}(CO)_{13}(PPh_3)_9]^{2+}$ clusters (blue). The spectrum was taken with an Orbitrap Exploris 240 mass spectrometer. S9

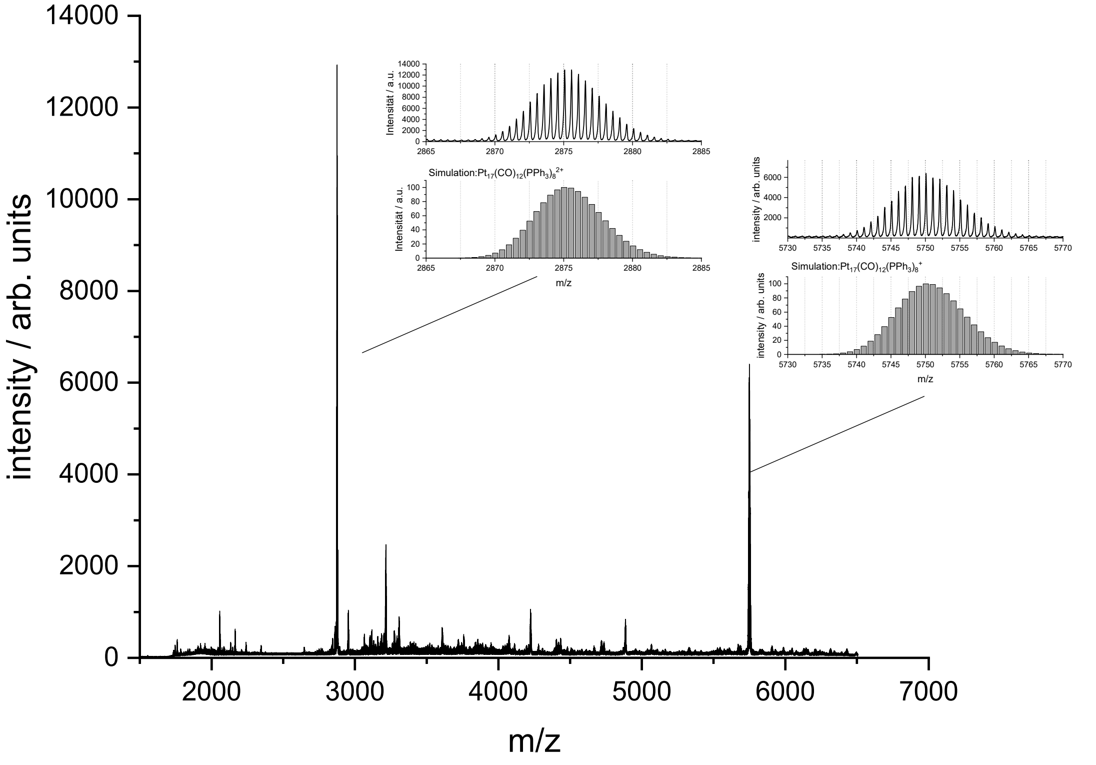

Figure S1. ESI-MS spectra of the reaction product at 120°C prior to purification. The major product observed was $[Pt_{17}(CO)_{12}(PPh_3)_8]^{n+}$ (n = 1, 2), with some $[Pt_{19}(CO)_{13}(PPh_3)_9]^{2+}$ as a minor by-product. The spectrum was taken with a timsTOF-MS.

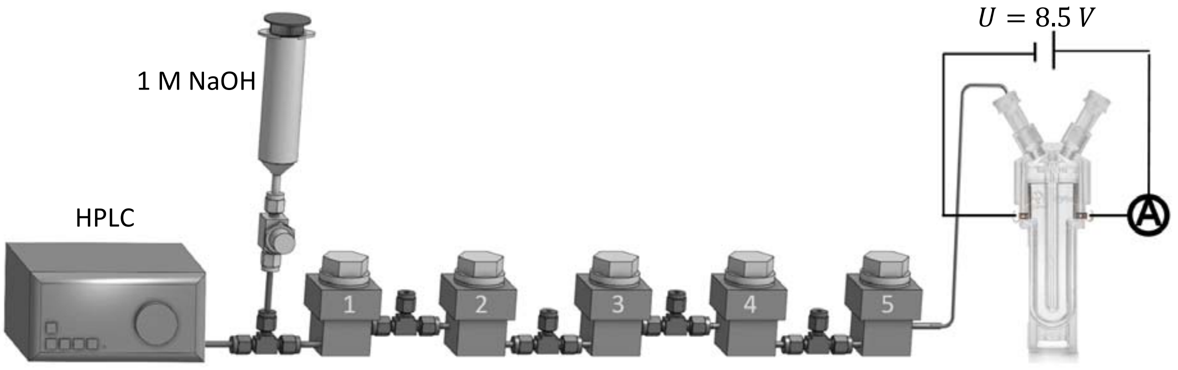

Figure S2. RTD measurement: A conductivity flow cell was used to record time-dependent concentration profiles at the outlet of each CSTR.

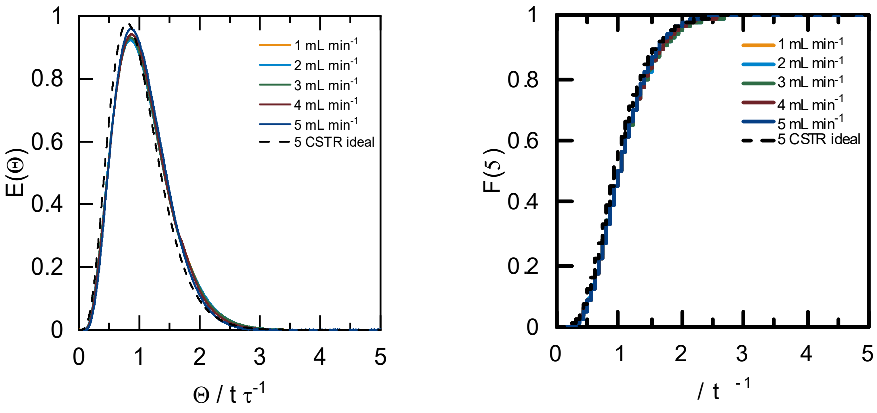

Figure S3. RTD profiles of five CSTRs in a cascade for different flow rates a) $E(\Theta)$ and b) $F(\Theta)$ (flow rates: 1 - 5 mL min^{-1}, stirring speed: 650 rpm; solid lines: experimental RTD profiles, dashed lines: RTD of the ideal CSTRs in series model, all normalized with $\Theta=t/\tau$).

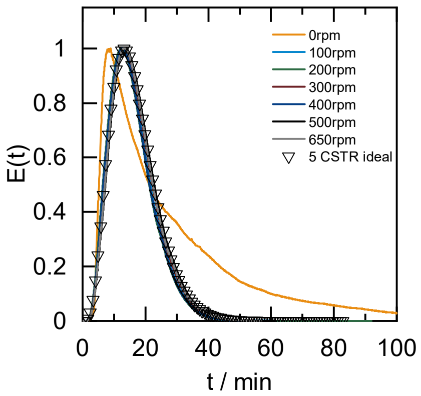

Figure S4. RTD profiles of five CSTRs in a cascade for different stirring speeds a) $E(t)$ (stirring speed: 0 and 650 rpm, flow rate: 3 mL min^{-1}, solid line: experimental RTD profile, triangles : RTD of the ideal CSTRs in series model).

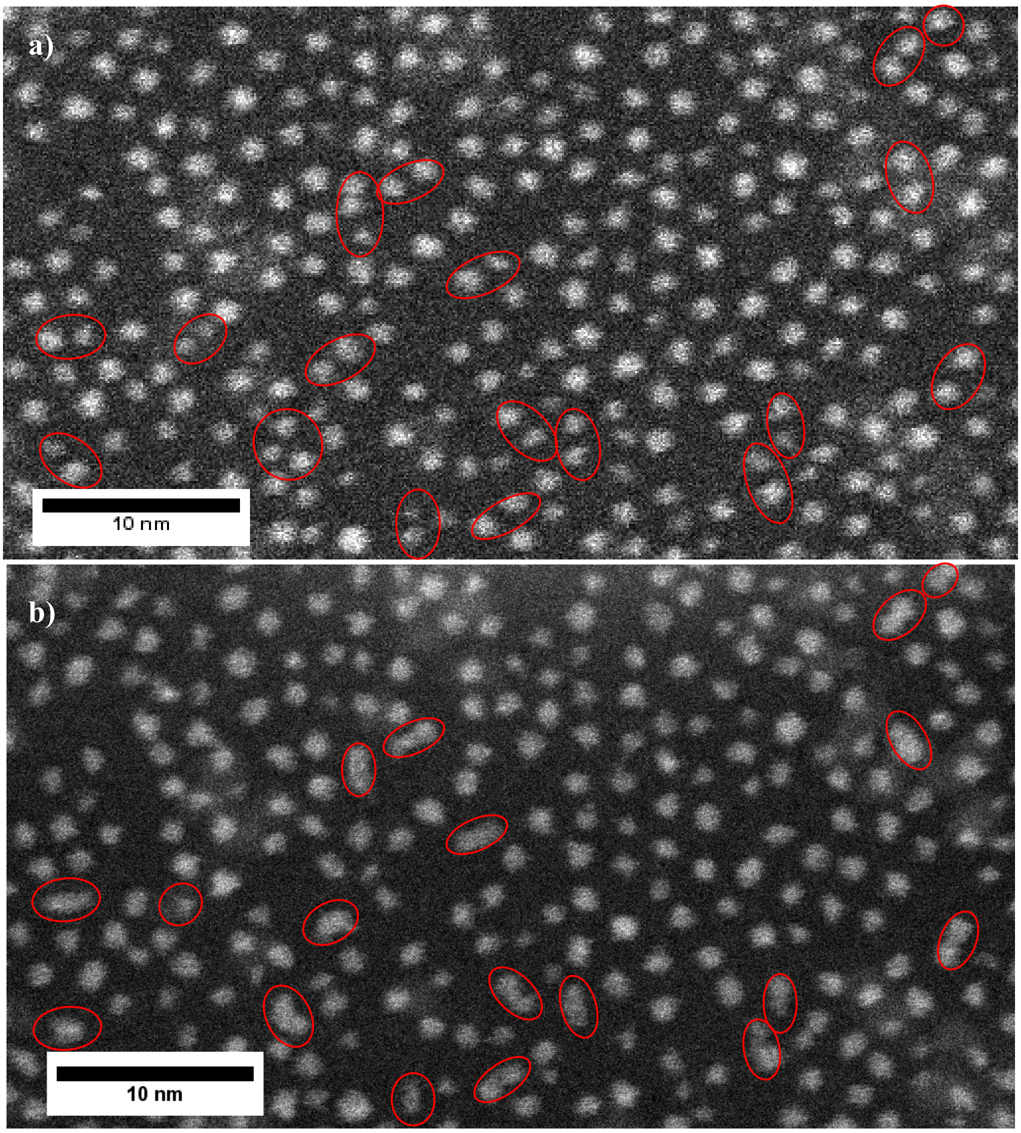

Figure S5. Room temperature STEM imaging: HAADF-STEM images of the $[Pt_{17}(CO)_{12}(PPh_3)_8]^{n+}$ (n = 1, 2) clusters showing the same area of the sample, but with some clusters merged in (b) due to damage caused by the electron beam. In (a), the region shown is cropped from an image captured at lower magnification and thus a lower dose rate ($0.38 \cdot 10^9$ electrons/sÅ^2). Image in (b) is acquired after the image in (a), and at a higher magnification and higher dose rate ($1.53 \cdot 10^9$ electrons/sÅ^2). Some of the clusters, marked here in red, have merged in the latter image. Sintering of clusters can in turn lead to an overestimation of the cluster size. In

order to decrease the effect of the electron beam and to more accurately measure the cluster dimensions, we also imaged the sample in a cryogenic holder as shown in the next image.

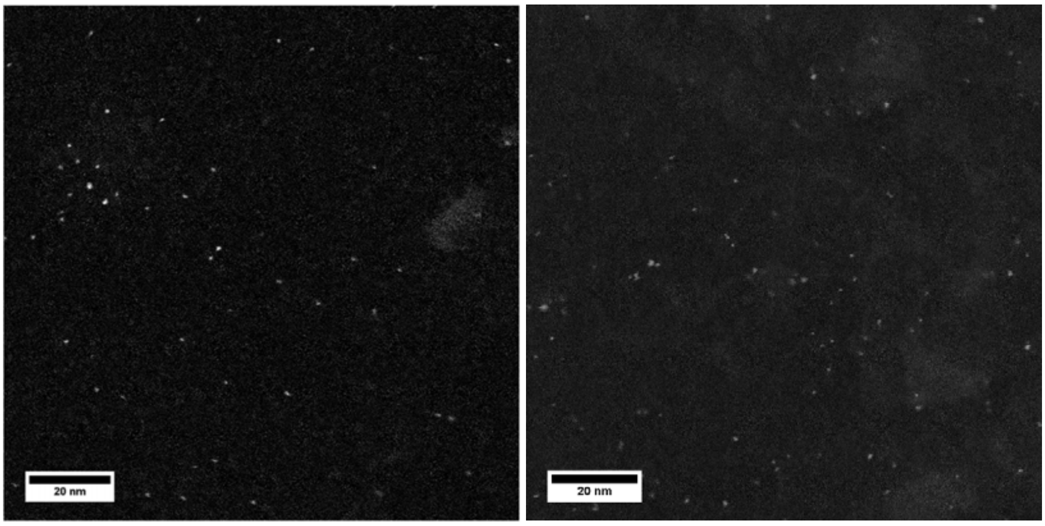

Figure S6. Low temperature STEM imaging: Low magnification HAADF-STEM images of the $[Pt_{17}(CO)_{12}(PPh_3)_8]^{n+}$ clusters, in a cryo holder at around -185 ºC, which were used for determination of the cluster size; mean size 1.09 ± 0.10 nm.

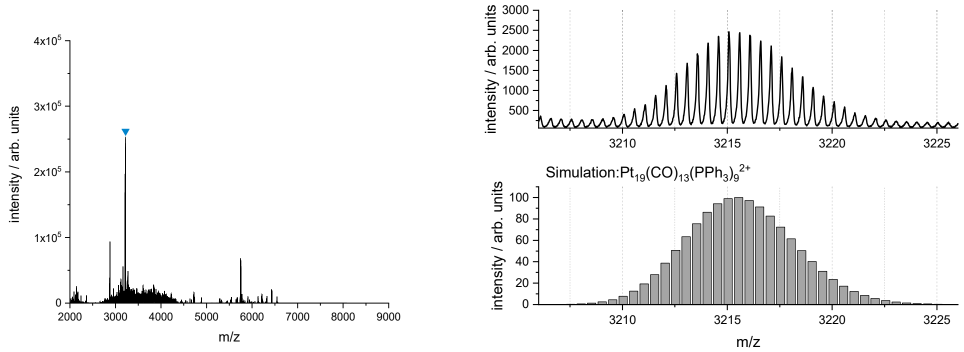

Figure S7. Experimental ESI-MS spectra of the reaction product after continuous flow synthesis at 125°C. Experimental and simulated ESI-MS spectra of $[Pt_{19}(CO)_{13}(PPh_3)_9]^{2+}$ clusters (blue). The spectrum was taken with an Orbitrap Exploris 240 mass spectrometer.
